# Feasibility of tailored treatment based on risk stratification in patients with early rheumatoid arthritis

**DOI:** 10.1186/s13075-014-0430-3

**Published:** 2014-09-25

**Authors:** Iris M Markusse, Jeska K de Vries-Bouwstra, K Huub Han, Peter AHM van der Lubbe, Anne A Schouffoer, Pit JSM Kerstens, Willem F Lems, Tom WJ Huizinga, Cornelia F Allaart

**Affiliations:** Department of Rheumatology, Leiden University Medical Center, PO BOX 9600, 2300 RC Leiden, the Netherlands; Department of Rheumatology, Maasstad Hospital, Rotterdam, the Netherlands; Department of Rheumatology, Vlietland Hospital, Schiedam, the Netherlands; Department of Rheumatology, Haga Hospital, the Hague, the Netherlands; Department of Rheumatology, Reade, Amsterdam, the Netherlands; Department of Rheumatology, VU Medical Center, Amsterdam, the Netherlands

## Abstract

**Introduction:**

Personalized medicine is the holy grail of medicine. The EULAR recommendations for the management of rheumatoid arthritis (RA) support differential treatment between patients with baseline characteristics suggestive of a non-poor prognosis (non-PP) or poor prognosis (PP) (presence of autoantibodies, a high inflammatory activity and damage on radiographs). We aimed to determine which prognostic risk groups benefit more from initial monotherapy or initial combination therapy.

**Methods:**

508 patients were randomized to initial monotherapy (iMono) or initial combination therapy (iCombo). Disease outcomes of iMono and iCombo were compared within non-PP or PP groups as determined on baseline characteristics

**Results:**

PP patients treated with iCombo after three months more often achieved ACR20 (70% vs 38%, *P* <0.001), ACR50 (48% vs 13%, *P* <0.001) and ACR70 response (24% vs 4%, *P* <0.001) than those treated with iMono, and had more improvement in HAQ (median decrease 0.75 vs 0.38, *P* <0.001). After 1 year, differences in ACR20 response and DAS-remission remained; PP patients treated with iCombo (vs iMono) had less radiographic progression (median 0.0 vs 1.5, *P* =0.001).

Non-PP patients treated with iCombo after three months more often achieved an ACR response (ACR20: 71% versus 44%, *P* <0.001; ACR50: 49% vs 13%, *P* <0.001; ACR70: 17% vs 3%, *P* =0.001) than with iMono, and functional ability showed greater improvement (median decrease in HAQ 0.63 vs 0.38, *P* <0.001). After 1 year, differences in ACR20 and ACR50 response remained; radiographic progression was comparable between the groups.

Non-PP and PP patients responded equally well to iCombo in terms of improvement of functional ability, with similar toxicity.

**Conclusions:**

Since PP and non-PP patients benefit equally from iCombo through earlier clinical response and functional improvement than with iMono, we conclude that personalized medicine as suggested in the guidelines is not yet feasible. The choice of treatment strategy should depend more on rapid relief of symptoms than on prognostic factors.

**Trial registration:**

Netherlands Trial Register NTR262 (registered 7 September 2005) and NTR265 (8 September 2005).

**Electronic supplementary material:**

The online version of this article (doi:10.1186/s13075-014-0430-3) contains supplementary material, which is available to authorized users.

## Introduction

Clinical trials have shown that on a group level, patients with early rheumatoid arthritis (RA) treated with initial combination therapy achieve earlier decrease in disease activity, improvement in functional ability and less radiographic joint damage progression than patients treated with initial monotherapy [1-7]. However, for individual patients there is a need for individualized treatment. The 2010 European League Against Rheumatism (EULAR) recommendations stated that ‘patients with a favourable prognosis very often respond similarly to low-intensity monotherapy or intensive medication strategiesʼ, suggesting that for patients with a poor prognosis this might be different [8]. It was also formulated that ‘occasional patients with a particular need for rapid, highly effective intervention, may benefit from starting a biological agent plus methotrexate as a viable and useful optionʼ, which was built on the idea that ‘patients with poor prognostic factors have more to gainʼ [8]. This opinion was abandoned in the updated 2013 recommendations, but these also state that ‘risk stratification is an important aspect of the therapeutic approach to RAʼ [9], detailing that after failure to achieve low disease activity on methotrexate monotherapy, ‘in patients with a low risk of poor RA outcome, another conventional synthetic disease-modifying antirheumatic drug (DMARD) strategy would be preferred, while in patients with a high risk, the addition of a biologic DMARD would be preferredʼ [9]. Hence, the recommendations encourage rheumatologists to use risk stratification in daily practice and to implement a personalized approach in the treatment of patients with RA.

In this post hoc analysis of the BeSt study, we investigated whether patients with poor or non-poor prognostic factors (based on previously developed prediction models [10-13]) respond differently to initial monotherapy, and whether patients with a poor or non-poor prognosis respond differently to initial combination therapy, as suggested by the EULAR recommendations. Furthermore, we studied the efficacy of a second conventional synthetic DMARD in patients with a low risk of poor RA outcome who failed on the first.

## Methods

### Patients

**Figure 1:**
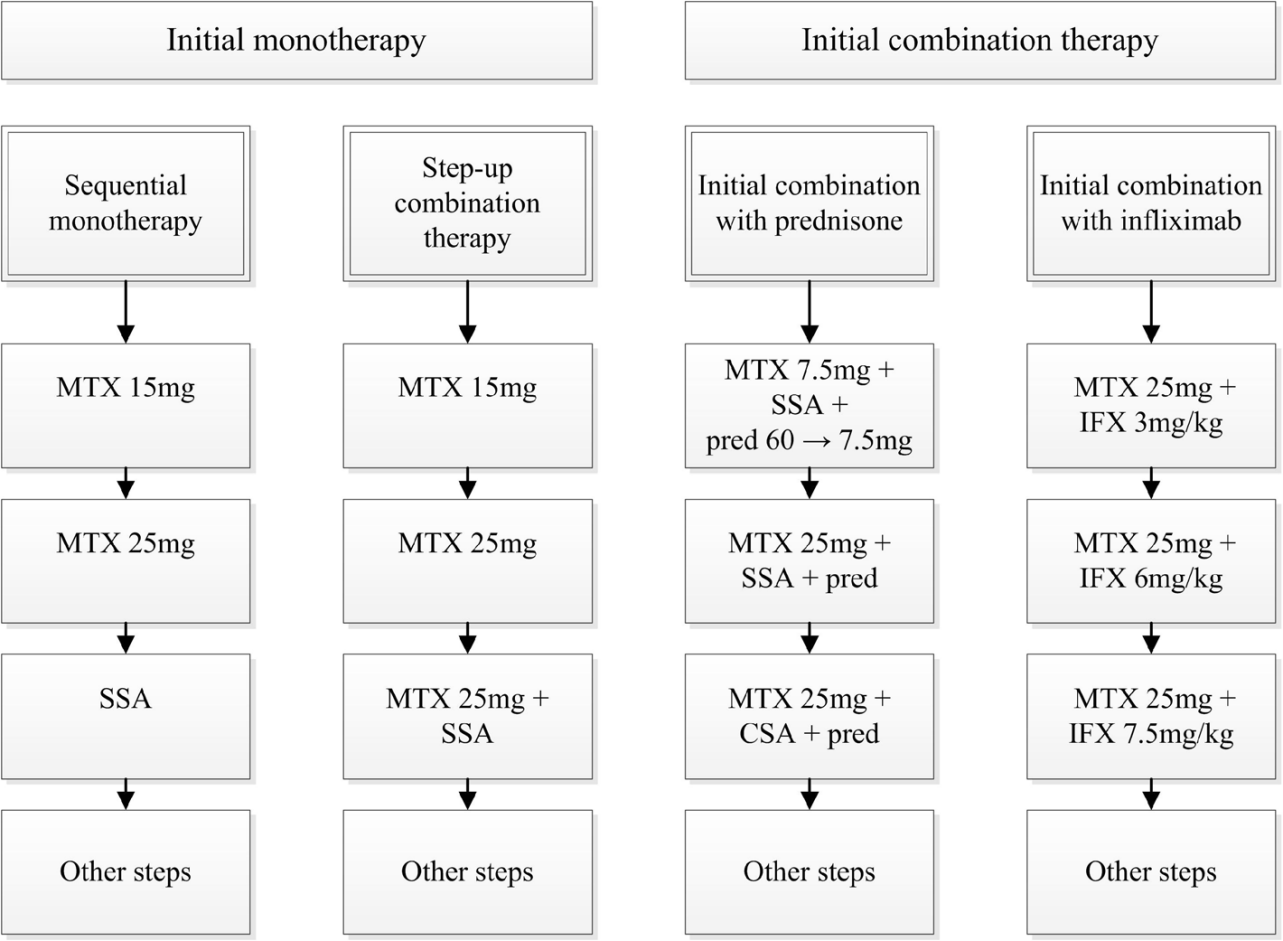
**Treatment steps per strategy.** CSA, ciclosporine A 2.5 mg/kg/day; MTX, methotrexate; IFX: infliximab; pred: prednisone 7.5 mg/day unless indicated otherwise; SSA, sulphasalazine 2000 mg/day.

In the BeSt (Dutch acronym for treatment strategies) study, 508 patients with early RA fulfilling the 1987 criteria [14] were included and randomized to one of four treatment strategies: (1) sequential monotherapy, (2) step-up combination therapy, (3) initial combination with methotrexate (MTX), sulfasalazine (SSA) and a tapered high dose of prednisone, (4) initial combination with MTX and infliximab. For this analysis, groups 1 and 2 (both starting with MTX monotherapy) were combined, because they had very similar disease outcomes during the first year of follow up [7], as also group 3 and 4 (both starting with combination therapy as shown in Figure 1). Three-monthly clinical assessments included the disease activity score (DAS) and the health assessment questionnaire (HAQ) to measure functional ability. Radiographs of hands and feet were collected yearly and assessed by two independent readers, in random order and blinded to patient identity, using the Sharp van der Heijde score (SHS) [15].

In all groups, the treat-to-target strategy required treatment adjustments when DAS was >2.4 (treatment steps are depicted in Figure 1). Dose tapering occurred if DAS was ≤2.4 for ≥6 months and the last antirheumatic drug was discontinued if DAS was <1.6 for ≥6 months (for details see previous publications [6,7]). The ethics committees of all participating centers approved the study protocol (listed in Acknowledgements) and patients gave written informed consent.

### Stratification for prognosis

Because there is no unambiguous method to determine which patients are ‘poor prognosis patients’ (PP patients), we used two different methods and tested both. The first method defined poor prognosis as presence of at least three out of four baseline disease characteristics, based on determinants used in prediction models [10-13]: DAS ≥3.7, swollen joint count (SJC) ≥10, erosions ≥4 and both rheumatoid factor (RF)-positive and anti-citrullinated peptide autoantibodies (ACPA)-positive. Consequently, non-poor prognosis patients (non-PP patients) were defined as having ≤2 features of poor prognosis. The latter category represents a heterogeneous group, including patients in a range from an evident favourable prognosis to patients with a moderate prognosis. The results of this stratification method are discussed in Results.

The second method was to classify all patients according to the matrix risk model for rapid radiographic progression (RRP, defined as an increase of ≥5 points in SHS during the first year) designed in the BeSt study [10]. This model estimates the risk of RRP with three baseline characteristics: the number of erosions, C-reactive protein and RF and ACPA status. Using the matrix for initial monotherapy, a cutoff of 50% risk for RRP was used to distinguish PP and non-PP patients. The results of this stratification method are shown in Additional files 1, 2, 3 and 4, and are not discussed in Results.

### Endpoints

Percentages of PP and non-PP patients treated with initial combination therapy who could discontinue prednisone or infliximab during the first year, because of a good response, were compared. Percentages of PP and non-PP patients receiving initial monotherapy who failed to achieve DAS ≤2.4 on MTX monotherapy after six months were compared, as well as percentages of DAS ≤2.4 three months after the introduction of a second conventional synthetic DMARD. To assess the outcomes of initial treatment options in non-PP and PP patients, we compared the clinical response (percentage of patients achieving DAS remission, defined as DAS <1.6 [16]; American College of Rheumatology (ACR)20, ACR50 and ACR70 response [17]; median decrease in HAQ) after three months and after one year. To define which patients benefit the most from initial combination therapy, the steepness of the slope of decrease in HAQ was compared between PP and non-PP patients. This was also tested for PP and non-PP patients receiving initial monotherapy. Radiographic progression (increase in SHS) at year one and the percentage of patients with RRP were compared between the groups. Adverse events (AE) and serious adverse events (SAE) were compared between PP and non-PP patients treated with initial combination therapy.

### Statistical analysis

The independent *t*-test, Mann-Whitney *U*-test, Fischer’s exact test, chi square (χ^2^) test, logistic regression analysis and linear regression analysis were used, depending on dichotomy or continuity and distributions of determinants and outcomes. For radiographic progression as the outcome, Poisson regression was used to take into account the non-normal distribution of radiographic progression, with an excess of zeros. To compare the decrease in HAQ between PP and non-PP patients, the mean difference was calculated and tested with the independent *t-*test. A *P*-value <0.05 was considered statistically significant.

## Results

Here, the results of defining PP patients by the presence of ≥3 of 4 poor prognostic factors (and consequently the non-PP patients by the presence of ≤2 of these factors) are discussed. The results of prognosis stratification according to the RRP matrix model of Visser *et al*. [10] are shown in Additional files 1, 2, 3 and 4.

Of 508 patients, 417 (82%) were classified as having a poor or a non-poor prognosis based on the available data. Of the 192/417 patients (46%) with PP, 100 (52%) had been randomized to initial monotherapy and 92 (48%) to initial combination therapy. Of 225/417 patients (54%) with a non-PP, 100 (44%) were treated with initial monotherapy and 125 (56%) with initial combination therapy.

Baseline characteristics per treatment strategy and prognosis category are shown in Table 1. Characteristics were similar among the randomization arms, but principally as a consequence of the stratification for prognosis, there were differences between prognosis categories. Although age was not a determinant to classify prognosis, patients with a poor prognosis were found to be older than patients with a non-poor prognosis.

**Table 1:** **Baseline characteristics of 417 patients classified as having a poor prognosis or a non-poor prognosis**

	**Poor prognosis patients**		**Non-poor prognosis patients**		
	**Initial mono**	**Initial combo**	**Initial mono**	**Initial combo**	***P*** **-value**
	**(n =100)**	**(n =92)**	**(n =100)**	**(n =125)**	
Age, years, mean ± SD	56 ± 13	58 ± 15	53 ± 13	51 ± 13	0.002
Gender, n (%) female	68 (68)	60 (65)	72 (72)	78 (62)	0.481
Treatment strategy, n (%)					<0.001
1. Sequential monotherapy (MTX)	51 (51)	0	54 (54)	0	
2. Step-up therapy (MTX)	49 (49)	0	46 (46)	0	
3. MTX, SSA and prednisone	0	43 (47)	0	61 (49)	
4. MTX and infliximab	0	49 (53)	0	64 (51)	
Disease activity score, mean ± SD	4.8 ± 0.7	4.5 ± 0.6	4.3 ± 0.9	4.1 ± 0.9	<0.001
Swollen joint count, median (IQR)	17 (11–22)	15 (12–18)	11 (8–16)	11 (8–17)	<0.001
Tender joint count, median (IQR)	14 (11–19)	13 (10–19)	13 (8–16)	12 (8–17)	<0.001
ESR, mean ± SD	51 ± 30	44 ± 29	37 ± 25	35 ± 24	<0.001
VAS gh, mean ± SD	61 ± 21	57 ± 22	60 ± 23	60 ± 21	0.754
HAQ, mean ± SD	1.4 ± 0.7	1.4 ± 0.7	1.3 ± 0.7	1.3 ± 0.7	0.633
RF-positive, n (%)	85 (85)	79 (86)	49 (49)	54 (43)	<0.001
ACPA-positive, n (%)	87 (89)	76 (84)	40 (41)	49 (40)	<0.001
Erosive disease, n (%)	79 (81)	78 (85)	62 (63)	74 (60)	<0.001

ACPA, anti-citrullinated autoantibodies; Erosive disease, defined as the presence of >0.5 erosion on radiographs of hands and feet; ESR, erythrocyte sedimentation rate; HAQ, health assessment questionnaire (0 to 3 scale); Initial combo, initial combination therapy with either prednisone or infliximab; Initial mono, initial monotherapy with methotrexate; MTX, methotrexate; Non-poor prognosis patients (presence of ≤2 of 4 poor prognostic factors); Poor prognosis patients (presence of ≥3 of 4 poor prognostic factors); RF, IgM rheumatoid factor; SSA, sulphasalazine; VAS gh, visual analogue scale (0 to 100 millimeter scale) of general health.

### Treatment response

Of 92 PP patients who received initial combination therapy, 47 (51%) could discontinue prednisone or infliximab after achieving low disease activity during at least six consecutive months. Similarly, of 125 non-PP patients treated with initial combination therapy, 70 (56%) could discontinue prednisone or infliximab (*P* =0.674).

After six months, 55/100 PP patients (55%) and 33/100 non-PP patients (33%) who had been allocated to initial monotherapy had not achieved a DAS ≤2.4 on MTX monotherapy despite a dose increase at three months from 15 mg/week to 25 mg/week (*P* =0.007). Three months later, 39/55 (72%) PP patients and 25/33 non-PP patients (76%) had also failed to achieve DAS ≤2.4 after switching to or adding SSA (*P* =0.364) and one non-PP patient (3%) was treated outside of protocol.

### Clinical outcomes after three months follow up

**Figure 2:**
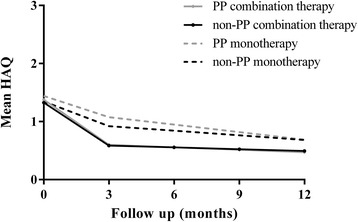
**Mean difference in health assessment questionnaire (HAQ) score in patients treated with initial combination therapy or initial monotherapy when prognosis was defined by prognostic factors.** HAQ scale 0 to 3; Non-PP, non-poor prognosis; PP, poor prognosis.

Significantly more PP patients who were treated with initial combination therapy fulfilled the ACR20 response criteria after three months than those treated with initial monotherapy (70% versus 38%, *P* <0.001). This was the same for ACR50 response (48% versus 13%, *P* <0.001), ACR70 response (24% versus 4%, *P* <0.001) and for DAS remission (17% versus 5%, *P* =0.016). Patients treated with combination therapy had a significantly greater improvement in functional ability (median decrease in HAQ 0.75 versus 0.38, *P* <0.001). This resulted in a mean HAQ score at 3 months of 0.60 in patients treated with initial combination therapy compared to a mean HAQ score of 1.08 in patients treated with initial monotherapy (see also Figure 2).

Non-PP patients treated with initial combination therapy more often met the ACR response criteria at three months compared to those treated with initial monotherapy; ACR20 (71% versus 44%, *P* <0.001), ACR50 (49% versus 13%, *P* <0.001) and ACR70 (17% versus 3%, *P* =0.001). They also showed more DAS remission (18% versus 7%, *P* =0.017) and a larger increase in functional ability (median decrease in HAQ score 0.63 versus 0.38, *P* <0.001). After three months, mean HAQ score was 0.59 in non-PP patients treated with initial combination therapy and 0.92 in those treated with initial monotherapy (see also Figure 2). In Table 2 the main results are summarized. With regression analyses similar results were obtained (data not shown).

**Table 2:** **Main clinical and radiographic outcomes of poor and non-poor prognosis patients receiving initial monotherapy or initial combination therapy after 3 months and after 1 year**

	**Poor prognosis patients**		
	**Initial mono**	**Initial combo**	***P*** **-value**
**DAS remission**			
After 3 months	5 (5)	15 (17)	0.016
After 1 year	21 (21)	31 (36)	0.034
**ACR20 response**			
After 3 months	35 (38)	57 (70)	< 0.001
After 1 year	73 (80)	75 (93)	0.026
**ACR50 response**			
After 3 months	12 (13)	40 (48)	<0.001
After 1 year	52 (57)	59 (71)	0.060
**ACR70 response**			
After 3 months	4 (4)	20 (24)	<0.001
After 1 year	28 (30)	35 (44)	0.081
**Decrease in HAQ score,** median (IQR)			
After 3 months	−0.38 (−0.63, 0.06)	−0.75 (−1.13, −0.25)	<0.001
After 1 year	−0.75 (−1.13, −0.38)	−0.88 (−1.38, −0.38)	0.110
**SHS progression**			
After 1 year, median (IQR)	1.5 (0, 5.0)	0 (0, 2.0)	0.001
RRP	24 (26)	8 (10)	0.006
	**Non-poor prognosis patients**		
	**Initial mono**	**Initial combo**	***P*** **-value**
**DAS remission**			
After 3 months	7 (7)	23 (18)	0.017
After 1 year	35 (36)	43 (36)	1.000
**ACR20 response**			
After 3 months	38 (44)	79 (71)	<0.001
After 1 year	63 (72)	96 (85)	0.024
**ACR50 response**			
After 3 months	12 (13)	56 (49)	<0.001
After 1 year	44 (52)	77 (68)	0.027
**ACR70 response**			
After 3 months	3 (3)	20 (17)	0.001
After 1 year	29 (33)	45 (39)	0.380
**Decrease in HAQ score,** median (IQR)			
After 3 months	−0.38 (−0.75, 0)	−0.63 (−1.13, −0.25)	<0.001
After 1 year	−0.63 (−1.13, −0.13)	−0.88 (−1.25, −0.31)	0.040
**SHS progression**			
After 1 year, median (IQR)	0 (0, 1.5)	0 (0, 1.0)	0.451
RRP	10 (11)	4 (4)	0.054

Numbers indicate number of patients (percentage) unless indicated otherwise. ACR response: according to the American College of Rheumatology criteria [17]; DAS remission, disease activity score <1.6 [16]; Initial combo: initial combination therapy with either prednisone or infliximab; Initial mono: initial monotherapy with methotrexate; non-poor prognosis (presence of ≤2 of 4 poor prognostic factors); HAQ, health assessment questionnaire (scale 0 to 3); poor prognosis (presence of ≥3 of 4 poor prognostic factors); SHS, Sharp van der Heijde score; RRP, rapid radiographic progression, defined as increase in Sharp van der Heijde score ≥5 points during the first year.

### Clinical and radiographic outcomes after one year follow up

Following initial combination therapy, after one year, PP patients more often achieved ACR20 response (93% versus 80%, *P* =0.026) and DAS remission (36% versus 21%, *P* =0.034) than following initial monotherapy. Other clinical outcomes were not significantly different after one year between PP patients treated with initial combination therapy or with initial monotherapy. Radiographic damage progression after one year was lower in PP patients treated with initial combination therapy than those treated with initial monotherapy (median (IQR) increase in SHS 0.0 (0.0 to 2.0) versus 1.5 (0.0 to 5.0), *P* =0.001) and there were significantly fewer patients with RRP (10% versus 26%, *P* =0.006).

After one year, more non-PP patients treated with initial combination therapy also fulfilled the ACR20 response criteria (85% versus 72%, *P* =0.024) and the ACR50 response criteria (68% versus 52%, *P* =0.027) than non-PP patients treated with initial monotherapy. Median (IQR) increase in SHS was 0.0 (0.0 to 0.5) in non-PP patients treated with initial combination therapy and 0.0 (0.0 to 1.0) in those treated with initial monotherapy (*P* =0.451). RRP occurred in 11% of non-PP patients treated with initial monotherapy compared to 4% in those treated with initial combination therapy (*P* =0.054). Table 2 shows a summary of these results. With regression analyses similar results were obtained (data not shown).

During the first year of follow up, the improvement in HAQ score after initial combination therapy was similar in PP and non-PP patients (*P* =0.795 after three months; *P* =0.687 after one year) (Figure 2). There was less improvement in HAQ score after initial monotherapy, again similarly in PP and non-PP patients (*P* =0.108 after three months; *P* =0.967 after one year).

### Toxicity

To evaluate possible toxicity of overtreatment with initial combination therapy, the numbers of PP and non-PP patients treated with initial combination therapy who reported an AE and/or SAE were compared. Of 92 PP patients randomized to initial combination therapy, 31 (34%) reported at least one AE or SAE, compared to 58/125 patients (46%) with a non-PP. Twenty-eight of 92 PP patients (30%) and 54/125 non-PP patients (43%) reported one or more AE (*P* =0.066). Four PP patients (4%) and six non-PP patients (5%) reported one or more SAE (*P* =1.000).

## Discussion

The results of this post hoc analysis in the BeSt study show that patients with recent-onset RA with a non-poor prognosis and patients with a poor prognosis respond similarly to the treatment strategy options. Both groups benefit more from initial combination therapy than from initial monotherapy and the success of a second conventional DMARD after failing on the first is limited in both groups.

Previous studies have shown that initial combination therapy results in better clinical and radiographic outcomes than initial monotherapy in patients with early RA on a group level [1-5,7]. It was suggested in the 2010 recommendations for the management of RA [8] that patients with favourable prognostic factors at baseline do not need initial combination therapy because they will respond equally well to initial monotherapy and that patients with a poor prognosis would benefit more from initial combination therapy. This was revoked in the 2013 update: it is now recommended that all patients should receive a similar initial treatment [9]. Still, the updated recommendations state that risk evaluation is an important aspect in the therapeutic approach of RA, and that patients with a favourable prognosis would require a different type of follow up treatment than patients with a poor prognosis after failure on initial MTX monotherapy [9].

To test these recommendations, we classified patients as having a poor prognosis (PP) or a non-poor prognosis (non-PP), as a representative of the heterogeneous group of patients ‘with a low risk of poor RA outcome’ mentioned in the updated 2013 recommendations, based on well-known and frequently used risk factors [10-13]. We tested whether these risk groups, over three-monthly evaluations in the first year of the BeSt study, responded differently to these treatments.

We found that in both PP and non-PP patients, initial combination therapy is more effective, compared to monotherapy, in inducing an early (that is, after three months) decrease in disease activity and early improvement in functional ability, this notwithstanding the fact that after six months on MTX monotherapy significantly more non-PP patients than PP patients achieved a low DAS (64% versus 43%). The improvement in functional capacity in patients treated with initial combination therapy was equal in PP and non-PP patients, both after three months and after one year. This indicates an early equal gain in functional capacity in both prognosis categories. These differences in clinical outcomes are explicit after three months, and remain, following treat-to-target adjustments in therapy, only marginal after one year.

There was no difference among PP and non-PP patients in response to SSA as the second conventional synthetic DMARD after failure to achieve a low DAS on initial MTX monotherapy: similarly low percentages of patients achieved a DAS ≤2.4 (21% of non-PP patients and 28% of PP patients). This appears to be at odds with recommendation 8 of the updated 2013 EULAR recommendations for the management of RA [9].

Overall, as a consequence of the definition of poor or non-poor prognosis, patients with a non-PP showed less radiographic joint damage progression than patients with a poor prognosis. After one year of targeted treatment, significantly less radiographic joint damage progression occurred after initial combination therapy in PP patients than after initial monotherapy. Thus it appears that for radiographic damage progression indeed, as originally formulated in the 2010 EULAR recommendations for the management of RA, PP patients ‘have more to gainʼ from the initial treatment choice [8].

Our definition of poor or non-poor prognosis was based on factors that are associated with (rapid) radiographic progression and are also used in prediction models [10-13]. However, early treatment initiation and targeted therapy, including the option of biologic DMARDs, have contributed to prevent this disease outcome in most BeSt patients to date. As RRP nowadays can also be better prevented with early effective treatment, models designed to predict RRP perform moderately in clinical practice. In addition, they do not provide information on clinical outcomes. Of the patients defined as PP according to the presence of ≥3 risk factors, only 26% actually developed RRP when treated with initial monotherapy and 10% developed RRP when treated with initial combination therapy. When PP is defined according to the matrix model of Visser *et al*. [10], 46% and 12% developed RRP when treated with initial monotherapy or combination therapy, respectively (Additional file 2). Thus, despite familiarity with prognostic factors, it is still difficult to predict the prognosis.

Consequently, it is proper to evaluate the efficacy of the initial treatment choice in terms of rapid relief of symptoms and functional improvement due to suppression of inflammation. Our data show that initial combination therapy is more successful in achieving these outcomes than initial MTX monotherapy, both for PP patients and for non-PP patients. In fact, clinical responses were very similar (and satisfactory) in all patients if they received initial combination treatment. In addition, although maybe not clinically relevant, PP patients showed less radiographic damage progression after initial combination therapy than after initial monotherapy. Also, more than half of the patients receiving initial combination therapy could discontinue prednisone or infliximab due to low disease activity, as soon as the protocol allowed drug discontinuation.

There was no significant difference in the number of AEs and SAEs reported by PP or non-PP patients on initial combination therapy. Similar toxicity among the four treatment arms has already been reported [6,7]. Hence, it appears that extra caution for the use of combination therapy in either group is not warranted.

## Conclusion

The definition of non-poor or poor prognosis shows a moderate performance in predicting radiographic progression, despite the use of two different methods and based on risk factors in validated prediction models. Overall, patients in the BeSt study benefitted from initial combination therapy with better clinical outcomes and more functional improvement at three months than after initial monotherapy, regardless of prognosis category. Response to a second conventional synthetic DMARD after failure on methotrexate monotherapy was similar in patients with a poor or a non-poor prognostic profile, and generally disappointing. These results suggest that prognostic factors associated with future radiographic damage progression contribute little to predict early clinical response to initial treatment, and therefore, in our opinion tailored treatment based on prognosis as suggested by the EULAR guidelines is currently not feasible. The choice of treatment strategy may depend less on these prognostic factors and more on the estimated need for rapid relief of symptoms and limitations due to active disease in our patients.

## Additional files

Additional file 1:
**Results.** File contains data about risk stratification based on the matrix model of Visser *et al*., on mean difference in health assessment questionnaire (HAQ) score and toxicity. Additional file 2: Table S1. File contains data about risk stratification based on the matrix model of Visser *et al*., on clinical and radiographic outcomes using the *t*-test and Fischer’s exact test. Additional file 3: Table S2. File contains data about risk stratification based on the matrix model of Visser *et al*., on clinical and radiographic outcomes using regression analyses. Additional file 4: Figure S1. File contains a figure showing the mean difference in health assessment questionnaire (HAQ) score after risk stratification based on the matrix model of Visser *et al*.

## Abbreviations

ACPA: anti-citrullinated protein antibody
ACR: American College of Rheumatology
AE: adverse event
DAS: disease activity score
DMARD: disease-modifying antirheumatic drug
EULAR: European League Against Rheumatism
HAQ: health assessment questionnaire
iCombo: initial combination therapy
iMono: initial monotherapy
MTX: methotrexate
non-PP: non-poor prognosis
PP: poor prognosis
RA: rheumatoid arthritis
RF: IgM rheumatoid factor
RRP: rapid radiographic progression
SAE: severe adverse event
SHS: Sharp van der Heijde score
SJC: swollen joint count
SSA: sulphasalazine
