# Supplementary material for: Feasibility of tailored treatment based on risk stratification in patients with early rheumatoid arthritis
Source: Arthritis Res Ther. 2014 Sep 25;16(5):430. doi: 10.1186/s13075-014-0430-3 (PMC4203912; doi:10.1186/s13075-014-0430-3)
Supplement: Additional file 1: — Results. File contains data about risk stratification based on the matrix model of Visser et al., on mean difference in health assessment questionnaire (HAQ) score and toxicity. [file 13075_2014_430_MOESM1_ESM.pdf]

ADDITIONAL FILE 1

Additional results

Poor prognosis (PP) or non-poor prognosis (non-PP) patients were defined by the matrix risk model for rapid radiographic progression, using a cut off of 50%. Using this method, 398 of 508 patients (78%) could be classified, of which 92 patients (23%) were defined as having PP. Of these, 44 received initial monotherapy and 48 initial combination therapy. Of 306/398 (77%) classified as having a non-PP, 144 were treated with initial monotherapy and 162 with initial combination therapy. Of the In additional table 1 and additional table 2, clinical responses after three months and after one year are shown.

During the first year, mean difference in HAQ was similar in PP and non-PP patients (p=0.506 for comparison after three months; p=0.551 for comparison after one year), as also shown in the additional figure.

In patients treated with initial combination therapy, results with regard to (severe) adverse events and discontinuation of prednisone or infliximab were similar (data not shown). Success rates of the introduction of sulphasalazine in patients who failed on methotrexate monotherapy were similar too (data not shown).
