# Supplementary material for: Feasibility of tailored treatment based on risk stratification in patients with early rheumatoid arthritis
Source: Arthritis Res Ther. 2014 Sep 25;16(5):430. doi: 10.1186/s13075-014-0430-3 (PMC4203912; doi:10.1186/s13075-014-0430-3)
Supplement: Additional file 4: Figure S1. — File contains a figure showing the mean difference in health assessment questionnaire (HAQ) score after risk stratification based on the matrix model of Visser et al. [file 13075_2014_430_MOESM4_ESM.pdf]

ADDITIONAL FILE 4

Additional figure 1: Mean difference in HAQ in patients treated with initial combination therapy or with initial monotherapy, when prognosis defined by the matrix model of Visser et al.

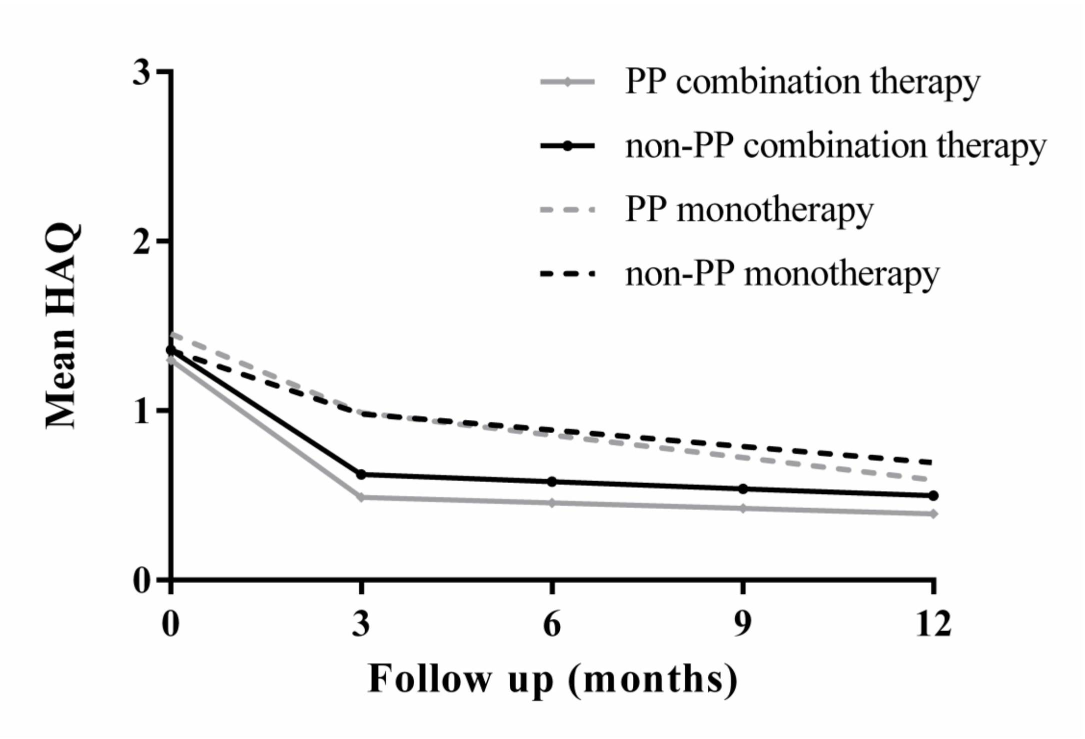

HAQ: health assessment questionnaire (scale 0 – 3).
